# Emerging Medical Therapies for the Treatment of Obesity in Women with Cardiovascular Diseases

**DOI:** 10.1007/s11886-023-01961-z

**Published:** 2023-10-24

**Authors:** Leili Behrooz, Carrie G. Lenneman, Naomi M. Hamburg

**Affiliations:** 1 Whitaker Cardiovascular Institute and Section of Vascular Biology, Boston University Chobanian and Avedisian School of Medicine, 72 East Concord St, Boston, MA 02118 USA; 2 University of Alabama at Birmingham, UAB Heersink School of Medicine, Birmingham, AL USA

**Keywords:** Obesity, Women, Cardiovascular disease, Risk factors, Prevention

## Abstract

**Purpose of Review:**

In this review, the impact of obesity on cardiovascular disease in women and emerging anti-obesity pharmacologic treatments are discussed.

**Recent Findings:**

Robust evidence demonstrates the burden of obesity across the lifespan in women and links obesity to a diverse set of cardiovascular diseases. Female-specific risk factors including sex hormones and pregnancy factors intersect with obesity and cardiovascular risk. Sustained weight loss has potential for cardiovascular benefits. Recent trials demonstrate cardiovascular benefits of emerging agents with weight loss effects including GLP-1 RA and SGLT2 inhibitors in women.

**Summary:**

Treatment and prevention strategies for cardiovascular disease in obese women should include integration of weight management strategies including the targeted use of emerging pharmacologic therapies.

## Introduction

**Fig. 1:**
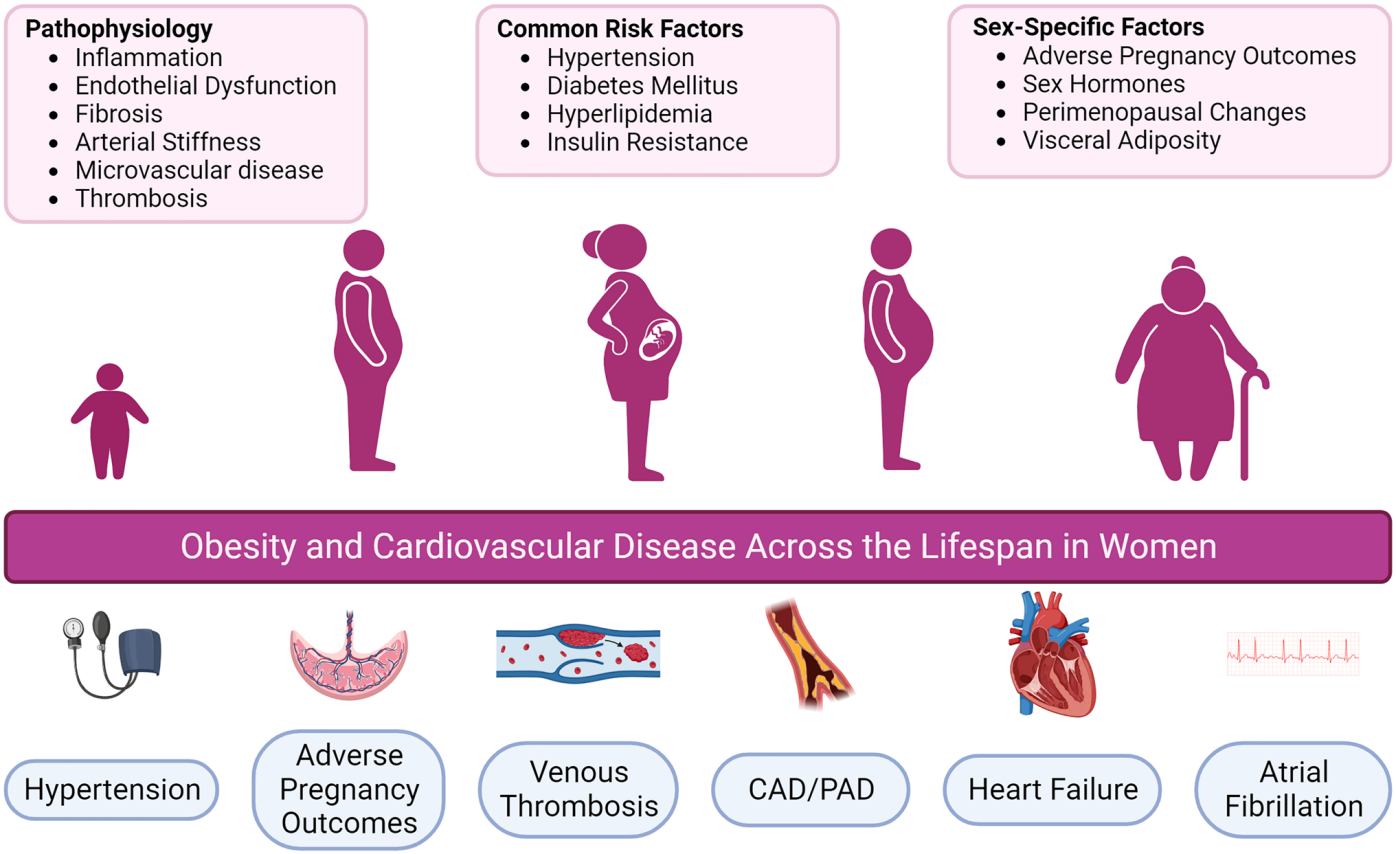
Impact of obesity on cardiovascular disease across the lifespan in women. Created with BioRender.com

Obesity contributes to the development of a diverse set of cardiovascular diseases. Understanding the burden of obesity and heart disease in women is critical to prevention efforts [1]. Women have high rates of obesity associated with a sex-based accentuation of cardiovascular risk. Importantly, obesity impacts women across the lifespan including in youth, pregnancy, the midlife transition, and in older age (Fig. 1) [2]. In the USA, more than 40% of adult women are obese [3, 4]. Worldwide, obesity leads to 2 million deaths in women, largely related to cardiovascular causes [5, 6]. Health disparities exist in obesity rates among women and in the presence of metabolically unhealthy obesity [7–9]. Chronic inflammation in obesity drives adverse systemic processes including vascular dysfunction, insulin resistance, metabolic dysregulation, hypertension, and fibrosis [10].

Shared pathophysiologic mechanisms between obesity and concomitant cardiometabolic conditions including type 2 diabetes (T2DM), hypercholesterolemia, chronic kidney disease, and hypertension suggest that similar therapies may be beneficial. Treatments initially developed for T2DM including glucagon-like peptide-1 receptor agonist (GLP-1 RA) medications and sodium-glucose transporter 2 (SGLT2) inhibitors now have proven benefits for obesity-related cardiometabolic diseases beyond glucose lowering. The intersection of obesity and cardiovascular disease in women heightens the importance of cardiovascular clinicians in the multidisciplinary management of weight loss therapies. The current review discusses obesity-related cardiovascular disease in women with a focus on emerging medication treatments to restore cardiometabolic health and promote weight loss.

## Impact of Obesity Across the Life Course in Women

### Epidemiology of Obesity in Women

Obesity has a disproportionate influence in women from youth to old age. Most recent US estimates indicate that 18% of female children and 41% of adult women are obese, of whom 11.6% have severe obesity [7]. Rates are higher in both youth and adult women who are Black compared to Non-Hispanic White, Hispanic, and Asian women [8]. Rural residence and a higher burden of unfavorable social determinants of health associate with higher obesity prevalence in women [11, 12]. Increases in severe obesity rates over the past 20 years are higher in women than men [13, 14]. Reflecting underlying population trends, pre-pregnancy obesity rates are also rising with close to a third of people being obese prior to pregnancy in 2019 [15]. During the menopausal period, women develop both higher overall body mass index (BMI) and a shift toward central obesity, a more metabolically toxic fat distribution pattern. The prevalence of obesity remains elevated in older women who are also at the greatest risk of cardiovascular diseases including atrial fibrillation, heart failure, and atherosclerosis.

### Pathophysiologic Connections of Obesity and Cardiovascular Disease in Women

A potential conceptual model linking obesity to cardiovascular disease in women includes higher prevalence of cardiometabolic risk factors that contribute to systemic inflammation, vascular dysfunction, and myocardial remodeling [16–19]. In addition, excess adipose tissue may produce factors that alter cardiovascular homeostasis [20•]. Genetic factors as well as gene-environment interaction are increasingly recognized as important drivers of obesity and adverse metabolic state [21, 22].

Sex differences in both traditional risk factors along with female-specific risk factors may be contributing to the accelerated risk of cardiovascular disease in obese women (Fig. 1) [23]. T2DM appears to confer a stronger risk of cardiovascular disease in women [24–27]. Obesity attenuates the relative protection from hypertension in younger women [28]. Fat distribution differs by sex with more visceral adiposity in women along with sex-based differences in the metabolic activity of ectopic fat deposits [20, 29–34]. Sex hormones, including estrogen, regulate adipose tissue biology, leading to a more unhealthy phenotype in the postmenopausal period [35]. Age-related arterial stiffening is greater in women as is insulin-resistance driven microvascular dysfunction [36–40]. Sex hormones also contribute to endothelial function with a greater decline in vasomotor function in women following menopause [41, 42]. Additional factors related to reproductive health may also contribute to sex-specific obesity risks including hypertensive disorders in pregnancy, infertility, and other pregnancy complications [43, 44].

## Association of Obesity and Weight Loss with Cardiovascular Diseases in Women

### Obesity and Hypertension in Women

Obesity accelerates the age-related rise in blood pressure [45, 46]. Overall, the presence of obesity is associated with a marked increased risk of incident hypertension in women [28]. Weight gain accelerates age-related increases in blood pressure [47]. Changes in the proportion of estrogen and androgens in the presence of visceral adiposity may also be important in driving obesity-related hypertension in women [16, 48]. A recent study focused on the menopause transition showed an association of higher BMI with both a pattern accelerated change in menopause and with a high blood pressure in the premenopausal period [49••]. Sex-based differences have also been demonstrated in vasodilator function particularly related to altered mineralocorticoid regulation in the endothelium [50, 51]. Weight loss with bariatric surgery produces marked improvement in blood pressure in the early period with attenuation over time [52]. Appropriate treatment of blood pressure is important in the overall management of women with obesity and hypertension.

### Obesity and Coronary Heart Disease and Peripheral Artery Disease in Women

A recent umbrella review of meta-analyses defined the risk of coronary heart disease with adiposity [53••]. An increase of BMI by 1 kg/m2 was associated with 4% higher risk of coronary disease in women compared to 6% in men. The presence of obesity in women was associated with a 1.58-fold higher risk of coronary heart disease. Obesity increases the lifetime risk of coronary disease by 85% in women with a 2.53-fold increase for severe obesity. PAD is more prevalent in women with obesity [54]. One study showed that BMI > 35 was associated with 1.35 odds of PAD and 2.98 odds of PAD for BMI > 40 in women suggesting that increasing BMI may be a strong independent risk factor for PAD in women [55]. Mendelian randomization confirms a causal association of adiposity with coronary disease indicating an overall 1.19 relative risk for each 5 kg/m2 increase in genetically predicted BMI [53••]. In addition to conduit vessel atherogenesis, excess adipose tissue may have particular adverse consequences in the microvasculature, a determinant of angina without obstructive coronary disease [56]. About half of the genetic-associated cardiovascular risk related to obesity is mediated by metabolic risk factors including lipids, hypertension, and T2DM [57]. A comprehensive assessment of circulating biomarkers demonstrated higher inflammatory markers and adipokines in women as compared to men [58•]. The constellation of associated risk factors with obesity emphasizes the importance of a comprehensive approach to therapies to mitigate atherosclerotic risk [59, 60]. In patients with T2DM, the rate of achieving risk factor control is lower in women as compared to men [61].

Weight fluctuations portend higher cardiovascular disease risk [62]. Dietary approaches to weight loss generally do not significantly alter coronary heart disease most likely related to the challenges of sustaining the weight loss [63]. In severe obesity, bariatric surgery has been shown to significantly decrease incident coronary artery disease [64, 65]. The greater impact of surgical weight loss is likely due to the lower degree of weight loss with lifestyle modifications vs. surgical interventions (5–10 kg vs. 10–40 kg) [66]. Also, sustained weight loss is more likely with surgical intervention which is important for achieving the beneficial effects of weight loss on atherosclerotic disease.

### Obesity and Heart Failure in Women

Both longitudinal observational and genetic studies indicate a causal association of obesity with incident heart failure in women [16, 67–70] failure in patients across the entire BMI spectrum which showed an increase in the prevalence of heart failure by 5% in men and 7% in women for each 1 kg/m^2^ increase in BMI [67]. Sex-differences are more pronounced in heart failure with preserved ejection fraction (HFpEF) [71, 72]. In a study of 4 community-based cohorts in the USA, obesity was most strongly associated with HFpEF in women as compared with men [70]. In the Women’s Health Initiative, obesity was an even more potent risk factor for HFpEF in Black American women compared with White women [73]. Inflammation, microvascular dysfunction, and insulin resistance are mechanisms common to obesity and HFpEF that may underlie sex-specific risk [70, 74–78]. Visceral adiposity, that is greater in women particularly following menopause, is associated with left ventricular remodeling and altered myocardial metabolism [79, 80]. Excess visceral adipose tissue was associated with altered exercise hemodynamics in women but not men with HFpEF [81•]. A recent meta-analysis suggested that in women, waist circumference and waist-to-hip ratio were superior predictors of heart failure risk in women pointing to a role for visceral adiposity [82]. Metabolic risk factors that travel with obesity are key drivers of heart failure risk in women consistent with the theme of comprehensive metabolic interventions [83].

In a bariatric surgery study including 77% women, surgical weight loss reduced heart failure rates compared to lifestyle intervention with a hazard ratio of 0.54 with a dose–response relationship between the degree of weight loss at 1 year [66]. In patients with pre-existing CVD, bariatric surgery lowered the rate of recurrent events in women with a HR of 0.6 [84]. New medications with impact on T2DM, obesity, and heart failure will be discussed in detail below.

### Obesity and Atrial Fibrillation in Women

Longitudinal studies confirm the importance of obesity as a risk factor for atrial fibrillation accounting for up to one-fifth of atrial fibrillation cases [85–87]. In the Women’s Heart Study, higher BMI was associated with heightened atrial fibrillation [85]. Obesity is associated with electrical remodeling in the atrial tissue including fibrosis and fat infiltration [88]. Measures of cardiac remodeling mediate the association of BMI with atrial fibrillation [19]. The association of BMI with atrial fibrillation is enhanced in women [89•].

Multiple randomized studies have evaluated the effect of weight loss on atrial fibrillation burden [90–92]. Consistently, weight management strategies combined with risk factor control reduce atrial fibrillation burden as well as improve results after ablation [93].

### Obesity and Venous Thromboembolism in Women

Obesity is a pro-inflammatory state, promoting a prothrombotic state. Prior studies show that obesity increases the risk of the first episode of venous thromboembolism (VTE) by 2.33 (95% CI, 1.68–3.24) [94]. Multiple adiposity measures including BMI, waist circumference, and fat percentage were associated with VTE risk in women but men [95••]. Epidemiologic study indicates that up to a quarter of VTE is attributable to elevated BMI in both women and men [96]. Moreover, obesity can increase the risk for VTE recurrence, which may be higher in women compared to men [97]. Hormonal treatments including oral contraceptives and hormone replacement therapy increase VTE risk that is enhanced with concomitant obesity [98]. In addition, VTE incidence is elevated during pregnancy, accounting for 9% of deaths during pregnancy in the USA [99, 100]. Higher BMI is an important risk factor for post pregnancy-related VTE [101].

### Obesity and Adverse Pregnancy Outcomes

With the rising prevalence of pre-pregnancy obesity, adverse pregnancy outcomes related to obesity are an important contributor to peri-pregnancy and long-term health in women [15, 44]. There are racial and ethnic disparities in the prevalence of pre-pregnancy obesity [102]. Both gestational diabetes and hypertension disorders of pregnancy have higher incidence with increasing BMI [103, 104]. Furthermore, high-risk obesity characterized by higher waist circumference confers a higher risk of adverse pregnancy outcomes [105•]. Robust epidemiologic evidence links adverse pregnancy outcomes to future development of cardiovascular risk factors and cardiovascular disease [106, 107]. A strategy to promote weight loss after pregnancy could reduce the direct and indirect risks of CVD [108]. A recent American Heart Association Scientific Statement addresses the importance of pre-pregnancy evaluation of obesity [44, 109].

## Approaches to Obesity Treatment in Women with CVD

### Overview of Anti-Obesity Strategies in Women with CVD

Treatment of obesity has the potential to improve cardiovascular risk factors and reduce the risk of cardiovascular events. Regular evaluation of BMI, cardiovascular risk factors, CVD, and readiness for weight loss is recommended. For individuals with BMI ≥ 30 or > 27 with CVD or risk factors, comprehensive lifestyle therapies are the first step in treatment [110, 111]. For individuals with BMI ≥ 40 or > 35 with CVD, evaluation for bariatric surgery is considered. For all patients with inadequate response to lifestyle therapy or with concomitant CVD, pharmacologic therapies can be considered. The backbone of all initial obesity therapies includes lifestyle and behavioral approaches including nutritional counseling and appropriate exercise augmentation. Similarly, weight loss is recommended for prevention of CVD using counseling and lifestyle approaches.

Studies suggest that to achieve cardiovascular benefits from weight loss in obesity, losing > 10% of body weight is needed [112]. Lifestyle changes with diet and physical activity may result in inadequate weight loss and may be complicated by weight regain [113]. Long-term studies have shown that bariatric surgical procedures typically lead to a sustainable weight loss of 25% and improvements in cardiovascular outcomes [65]. However, surgical approaches are an invasive method that is mainly reserved for cases of severe obesity [111].

There have been recent advances in the development of anti-obesity medications that are altering the treatment landscape. Studies with novel pharmacotherapy approaches for the treatment of obesity are now showing the degree of weight loss that may be relevant to altering CVD course. The focus of the following sections is on recent developments in medication treatment for obesity that is relevant to women with CVD. Earlier medications including orlistat, phentermine, lorcaserin, and naltrexone/buproprion have been reviewed previously, thus they are not the focus of the current review [114, 115].

### Glucagon-Like Peptide-1 Receptor Agonists and CVD in Women

Glucagon-like peptide-1 receptor agonist (GLP-1 RA) medications were developed and approved as glucose lowering agents in patients with T2DM [116]. Clinical trials in patients with T2DM demonstrated modest weight loss with all GLP-1 RA as shown in Table 1. The degree of weight loss seems largely related to dosing but may also reflect the extent of brain uptake and action. Both liraglutide and semaglutide have been approved for weight loss based on a series of trials in patients with obesity (Table 1). In the STEP trials, semaglutide resulted in an average weight loss of 12% by week 28 [117••]. The combination of liraglutide with an exercise intervention had enhanced efficacy for maintaining weight loss [118]. A newer T2DM treatment agent, tirzepatide, that combines GLP-1 RA with glucose-dependent insulinotropic polypeptide (GIP) activity, led to a greater degree of weight loss [119••]. In patients with BMI ≥ 30 or ≥ 27 with a risk factor, tirzepatide led to up to 20.9% weight loss with the highest dose.

**Table 1:** Selected clinical trials of GLP-1 RA and SGLT2 inhibitors relevant to obesity and CVD

	**Study population**	**% women**	**Study endpoint**	**Key findings**	**Sex-specific findings**
**GLP-1 RA**					
**T2DM + CVD outcome**					
ELIXA (lixisenatide) [120]	T2DM + recent ACS	30	MACE	HR 1.02 (0.89–1.17), − 0.6 kg weight loss	Not reported
LEADER (liraglutide) [121]	T2DM + CVD or RF	36	MACE	HR 0.87 (0.78–0.97), reduction in kidney dz progression, − 2.3 kg weight loss	HR 0.88 (0.72–1.08) P for interaction 0.84
SUSTAIN-6 (semaglutide) [122]	T2DM (83% with CVD)	39	MACE	HR 0.74 (0.58–0.95), − 4.3 kg weight loss 1.0 mg	HR 0.84 (0.54–1.31) P for interaction 0.45
HARMONY (albiglutide) [123]	T2DM + CVD	30	MACE	HR 0.78 (0·68–0·90), atrial fibrillation 0.82 (0.64–1.06)	HR 0.67 (0.50–0.89) P for interaction 0.23
REWIND (dulaglutide) [124]	T2DM + CVD or CVD RF	46	MACE	0.88 (0.79–0.99), − 1.46 kg weight loss	0.85 (0.71–1.02) P for interaction 0.60
PIONEER-6 (oral semaglutide) [125]	T2DM + CVD or CKD	32	MACE	0.79 (0.57–1.1)	Not reported
AMPLITUDE-O (efpeglenatide) [126]	T2DM + CVD or CKD	33	MACE	0.73 (0.58–0.92), − 2.6 kg weight loss	0.56 (0.36–0.86) P for interaction = NS
SELECT trial [127]	Obesity + CVD		MACE	News report 20% reduction	
**Obesity**					
STEP-1 (semaglutide) [128]	Obesity/overweight with RF	73	Weight loss	− 14.9% vs − 2.4% active vs. placebo, *P* < 0.001	Not reported
SCALE (liraglutide) [129]	Obesity/overweight with RF	78	Weight loss	− 5.6 kg, *P* < 0.001	Not reported
**GLP-1 RA + GIP**					
**Obesity**					
SURMOUNT-1 (tirzepatide) [119••]	Obesity/overweight with RF	67	Weight loss	− 15% (5 mg), − 19.5% (10 mg), − 20.9% (15 mg) vs. − 3.1% (placebo), all *P* < 0.001	Not reported
**SGLT2 inhibitor**					
**T2DM: CVD outcome**					
EMPA-REG (empagliflozin) [130]	T2DM + CVD	28	MACE	0.86 (0.74–0.99), − 2 kg weight loss	*P* = 0.81 for interaction
CANVAS (canagliflozin) [131]	T2DM + CVD or RF	36	MACE	0.86 (0.75–0.97), − 1.6 kg weight loss	0.84 (0.66–1.06), P for interaction 0.26
DECLARE-TIMI 58 (dapagliflozin) [132]	T2DM + CVD or RF	37	MACE	0.93 (0.84–1.03), − 1.8 kg weight loss	0.80 (0.68–0.94) *P* = 0.77
VERTIS CV (ertugliflozin) [133]	T2DM + CVD	30	MACE	0.99 (0.88–1.12), − 2 kg weight loss	0.90 (0.68–1.18)
SCORED (sotagliflozin) [134]	T2DM + CVD, CKD or RF	44	MACE	0.77 (0.65 − 0.91)	0.77 (0.60–0.99)
**Heart failure**					
DAPA-HF (dapagliflozin) [135]	CHF EF ≤ 40%	23	CV death or HF hosp	0.74 (0.65–0.85)	0.79 (0.59–1.06)
EMPEROR-reduced (empagliflozin) [136]	CHF EF ≤ 40%	23	CV death or HF hosp	0.75 (0.68–0.86)	0.59 (0.44–0.80)
EMPEROR-preserved (empagliflozin) [137]	CHF, EF > 40%	44	CV death or HF hosp	0.79 (0.69–0.90)	0.75 (0.61–0.87)
SOLOIST-WHF (sotagliflozin) [138]	T2DM + CHF hosp	33	CV death or HF hosp	0.67 (0.52–0.85)	Not reported
DELIVER (dapagliflozin) [139]	CHF, EF > 40%	44	CV death or HF hosp	0.82 (0.73–0.92)	0.82 (0.71–0.96)

Given the FDA requirement to evaluate cardiovascular safety of T2DM treatments, there have been dedicated cardiovascular studies for all GLP-1 RA medications [140]. All the cardiovascular outcome studies evaluated composite outcomes of MACE (cardiovascular death, myocardial infarction, and stroke) and enrolled patients with T2DM with a varying prevalence of pre-existing CVD. As shown in Table 1, selected GLP-1 RA’s demonstrated superiority compared to placebo for reduction of MACE. Meta-analyses confirm the benefits of GLP-1 RA’s in reducing individual endpoints including myocardial infarction, stroke, cardiovascular mortality, and all-cause mortality with only moderate heterogeneity across the agents [141, 142]. Among patients with T2DM, the benefit appeared similar for MACE events in individuals with established CVD compared to high burden of risk factors [142]. There was also a suggestion of reduction in hospitalization for heart failure though numbers of events were low and in heart failure with reduced ejection fraction, liraglutide did not show benefits [141]. It seems possible that a greater extent of cardiovascular benefit from GLP-1 RA medications is from the impact on atherosclerotic events. A recent trial (AMPLITUDE-O) looked at the effect of GLP-1 RA, efpeglenatide, for reducing the risk of cardiovascular events in patients with T2DM and CVD and was shown beneficial in reducing MACE compared to placebo [143]. The ongoing SELECT trial is evaluating semaglutide in patients with established CVD without T2DM and preliminary favorable results were reported in a press release [127]. Additional ongoing studies include in patients with atrial fibrillation as well as cardiovascular outcomes with tirzepatide. Multiple potential mechanisms have been proposed for the GLP-1 RA medications including direct vascular effects, alteration of risk factor profile, reduction of inflammation, and plaque stabilization [144–146].

Inclusion of women in the cardiovascular outcome trials of GLP-1 RA medications in patients with T2DM ranged from 30 to 46% and reporting on sex-specific results has not been consistent. One meta-analyses indicated similar benefits in women with T2DM with a HR of 0.88 (0.79–0.99, *P* = 0.03) for MACE compared to placebo [147••]. Another study using registry medication data sources suggested a higher degree of relative risk reduction with GLP-1 RA in women compared to men [148]. In the obesity trials with GLP-1 RA’s, the proportion of women has been higher consistent with many studies of weight loss interventions. In the STEP studies, more than 70% were women, but there is limited information about sex-specific effects. In the SURMOUNT-1 trial with tirzepatide, women comprised 67% of the study participants, but no information was provided in the initial report regarding sex-specific weight loss effects.

The most common adverse effects of GLP-1 RA medications include nausea, vomiting, and diarrhea [149]. In many patients, the intensity is mitigated by gradual dose escalation. The safety during pregnancy is not known and thus not recommended to be used during pregnancy. In general for both T2DM and for obesity management, GLP-1 RA represents a chronic therapeutic agent with evidence of weight regain when the medications are stopped.

### Sodium-Glucose Transporter 2 Inhibitors and CVD in Women

Similar to GLP-1 RA medications, sodium-glucose transporter 2 (SGLT2) inhibitors were developed for treatment of T2DM and have expanded into cardiovascular health impact. The role in obesity management is more variable [150, 151]. In trials of patients with T2DM, SGLT2 inhibitors reduce body weight by 1.5–2 kg in a dose-dependent manner that appears maintained over 4 years [152•]. Weight loss with SGLT2 inhibitors appears similar for patients without T2DM [153]. The SGLT2 inhibitor-induced weight loss is much more pronounced when combined with GLP-1 RA suggesting that the combination of glycosuria with appetite suppression may have additive benefits in patients with T2DM [154, 155].

There is robust evidence supporting the use of SGLT2 inhibitors to reduce cardiovascular risk in patients with T2DM and for the treatment of heart failure. Multiple agents demonstrate favorable cardiovascular outcome trials of SGLT2 inhibition in patients with T2DM as shown in Table 1. In meta-analyses, there is reduction of MACE only in patients with established atherosclerotic disease and heart failure outcomes in those with and without established CVD [156•]. A subsequent meta-analysis suggested primary MACE prevention in patients with CKD [157]. Subsequent trials have shown benefit in stable patients with heart failure with reduced and with preserved ejection fraction as well as in patients with chronic kidney disease (Table 1). Studies have shown that SGLT2 inhibitors improve cardiovascular outcomes and HF symptoms in patients with heart failure and preserved ejection fraction (HFpEF) and obesity, and this improvement is accompanied by weight loss [158]. Ongoing trials are evaluating outcomes with unstable coronary disease along with heart failure.

In patients with T2DM, the CV benefits of SGLT2 inhibitors appear similar in women compared to men. In a patient level meta-analysis of the dapagliflozin trials in heart failure for both reduced and preserved ejection fraction, there were no observed sex-based differences in the treatment benefits with a HR for women of 0.74 (0.66–0.84) [159]. Current guideline recommendations for both T2DM with CVD and for heart failure include the use of SGLT2 inhibitors [160]. Potential adverse effects of SGLT2 inhibitors include urinary tract infections and genital mycotic infections in women. The impact during pregnancy is not known and, thus, not recommended. Prior reports suggesting an increase in amputation risk have largely been refuted by more recent evidence suggesting safety in patients with peripheral artery disease [161]. It is important to note that SGLT2 inhibitors are not approved for weight management but for treatment of T2DM and heart failure though the combination with GLP-1 RA for patients with T2DM may augment weight loss.

## Incorporating the Treatment of Obesity in Cardiovascular Care of Women

Access to effective weight loss management strategies remains low even among patients with established CVD who may gain the greatest clinical benefits. Barriers include higher cost of medication therapies as well as fragmentation of care of patients with obesity. Multidisciplinary cardiometabolic management will be necessary to augment guideline-based use as appropriate. In the setting of cardiovascular practice, it is important to evaluate BMI for all patients as well as assess readiness for weight management strategies [162]. Cardiovascular clinicians are often the primary provider for patients with obesity and CVD [163•]. For patients with T2DM, the most recent ADA guidelines recommend GLP-1 RA medications added to metformin for patients with established atherosclerotic CVD or high risk indicators for CVD [164]. In patients with chronic coronary artery disease and T2DM, current AHA/ACC guidelines recommend GLP-1 RA or SGLT2 inhibitors as Class I [165]. Currently, estimates of implementation based on these recent guidelines remain low [166]. Weight reduction is greater with GLP-1 RA as compared to SGLT2 inhibitors [167]; however, patients with heart failure and chronic kidney disease have more direct benefit from SGLT2 inhibitors [156, 168]. Combination therapy with both agents may also be used in patients with need for heart failure, weight loss, and kidney protection [169, 170].

A recent study shows increasing prevalence of cardiologists prescribing SGLT-2 inhibitors (12-fold increase) and GLP-1 Ras (fourfold) from 2015 to 2020; however, overall total use by cardiologists remains low [171]. Incorporation of approaches to address cost concerns is also critical to prevent accentuation of health disparities [172]. Inclusion of anti-obesity medication therapies as a strategy for secondary prevention is an emerging facet of comprehensive cardiovascular care.

## Conclusions

The global rise in obesity prevalence is a major risk public health problem that affects women throughout their lifespan. Despite the strong association of obesity and CVD and the effect of weight loss on improving cardiovascular outcomes, obesity remains an undertreated condition. This review emphasizes the cardiovascular benefits of emerging therapies with an impact on obesity and approaches to incorporate use in appropriate patients with CVD. Ongoing clinical trials will provide additional information about the cardiovascular health impacts of GLP-1 RA and SGLT2 inhibitors as well as direct impact in patients with established CVD. Overall, there is robust evidence to support the inclusion of pharmacologic agents for weight management in the care of women with cardiovascular diseases.
